# Mechanical, microstructural, and free-vibration characteristics of concrete reinforced with recycled E-waste PVC fibers

**DOI:** 10.1038/s41598-026-44699-8

**Published:** 2026-03-20

**Authors:** Muluken Bogale Admasu, Bisrat Gissila, Andinet Aklilu, Hewan Dejene

**Affiliations:** 1 Faculty of Civil Engineering, Arba Minch Institute of Technology, Arba Minch University, P.O. BOX 21, Arba Minch, Ethiopia; 2 Department of Construction Technology and Structural Materials, RUDN University, Moscow, 117198 Russia

**Keywords:** Concrete, Recycled polyvinyl chloride fibers, Free-vibration characteristics, Mechanical properties, Damping ratio, Engineering, Materials science

## Abstract

This study investigates the influence of recycled polyvinyl chloride fibers derived from electronic waste on the mechanical and free-vibration characteristics of concrete. Fibers of two lengths (30 mm and 50 mm) were incorporated at contents of 0.8%, 1.0%, and 1.2%, and their effects on compressive strength, flexural strength, and free-vibration response were evaluated. Scanning electron microscopy was employed to examine fiber-matrix interactions and associated energy dissipation mechanisms. Results indicate that a fiber content of 0.8% yields the highest compressive strength, while higher contents result in slight reductions, which may be attributed to fiber agglomeration and increased porosity. In contrast, flexural strength increases progressively with fiber content, reaching a maximum of 22.1% at 1.2%, with longer fibers (50 mm) providing additional enhancement due to improved crack-bridging capacity. Free-vibration testing reveals a maximum increase in the damping ratio of approximately 7.47%, primarily governed by fiber content, with fiber length exerting a comparatively minor influence. Microstructural observations reveal strong interfacial bonding accompanied by localized debonding and controlled fiber pull-out, which likely contribute to enhanced energy dissipation. These findings suggest that recycled polyvinyl chloride fibers can serve as a sustainable reinforcement strategy for improving the free-vibration performance of concrete.

## Introduction

Concrete structures are frequently subjected to dynamic actions such as earthquakes, wind loads, vehicular traffic, and mechanical impacts, which may accelerate structural deterioration or, in severe cases, lead to failure. To mitigate these effects, previous studies have generally adopted two complementary approaches: the use of external vibration control devices and the enhancement of the intrinsic damping capacity of structural materials^1, 2, 3^. Improving the inherent damping of concrete is particularly attractive because it can enhance structural performance without requiring additional space, complex installation, or extensive maintenance compared with external control systems^4^.

To improve both mechanical and dynamic performance, various modifiers such as steel fibers, rubber particles, silica fume, and glass fiber have been incorporated into concrete^5^–^8^. In recent years, increasing attention has shifted toward sustainable alternatives, particularly recycled fibers derived from waste streams. A comprehensive review by Li et al.^9^, highlighted that recycled textile and fiber wastes, including glass, carbon, polymer, and metal fibers, can enhance crack control and mechanical performance while contributing to waste reduction. However, the effectiveness of such fibers strongly depends on fiber-matrix interfacial behavior, as weak bonding may limit stress transfer and energy dissipation^10^.

Parallel to these developments, numerous studies have explored broader sustainable reinforcement strategies. These include low-cost fiber-reinforced polymer systems for improving reinforced concrete members^11^, sustainable composites for lightweight aggregate concrete^12^, and strengthening techniques for masonry systems under diverse loading conditions^13, 14^. In addition, the development of alternative binders such as calcium sulfoaluminate cement derived from industrial by-products illustrates the ongoing transition toward low-carbon construction materials^15^. Collectively, these studies establish a broader framework for exploring recycled polymer fibers as sustainable reinforcement in concrete.

The rapid accumulation of discarded electrical cables has created an urgent need for the circular use of materials. Recycled polyvinyl chloride (PVC) fibers extracted from electric wiring have shown promise in improving the compressive and flexural performance of concrete^16, 17, 50^. Studies by Gull and Balasubramanian^18^ and Varghese and Boby^19^ reported optimal mechanical performance at fiber dosages near 0.8–1.0%, although these investigations primarily focused on static strength. While these studies demonstrate promising mechanical benefits, understanding the suitability of PVC fibers for vibration-sensitive structures requires examining their influence on damping behavior.

Previous research indicates that damping behavior in fiber-reinforced concrete varies considerably with fiber type and dosage. For example, steel fibers have been shown to increase the damping coefficient, with optimal performance reported at approximately 2% fiber content and 50 mm fiber length^20, 21^. In contrast, studies on polypropylene fibers report that relatively small fiber dosages can produce measurable damping improvements, while increasing the fiber content beyond certain levels may lead to a reduction in damping response^22, 23^. Glass fibers have also demonstrated potential to enhance damping behavior, although a clear optimal dosage has not yet been established^4,^^7^. The ductile nature of PVC fibers and their potential for interfacial sliding and pull-out suggest that they may contribute to enhanced energy dissipation. However, the dynamic behavior and damping characteristics of PVC fiber-reinforced concrete remain insufficiently explored^24, 25, 26^.

Accurate characterization of this behavior requires reliable measurement techniques. Conventional damping measurement methods, such as ASTM E756, were originally developed for homogeneous and lightly damped materials^27, 28, 29^. Recent studies have extended these approaches to fiber-reinforced cementitious composites^7^^,30^–^32^. Building on these developments, the present study employs a free-vibration attenuation method using a low-cost experimental setup based on an MPU6050 MEMS accelerometer integrated with an Arduino-based data acquisition system.

Accordingly, this study investigates the influence of recycled PVC fibers on the mechanical and dynamic behavior of concrete. By evaluating the effects of fiber content, fiber length, and beam span through mechanical testing, free-vibration analysis, and scanning electron microscopy (SEM), this research provides new insights into the potential of e-waste-derived reinforcement for vibration-sensitive structural elements such as industrial floors, foundations, and other dynamically loaded infrastructure. In particular, the study provides an integrated experimental assessment linking fiber characteristics, microstructural observations, and measured damping response, which has not been systematically reported for recycled PVC fiber-reinforced concrete.

## Experimental method

### Materials

Ordinary Portland cement (OPC) of strength class 42.5 R was used as the primary binder in this study, targeting a characteristic cube compressive strength of 35 MPa. Natural river sand with a fineness modulus of 2.9 and a maximum particle size of 4.75 mm was employed as the fine aggregate, conforming to relevant standards for concrete production. Crushed stone with particle sizes ranging from 5 mm to 19 mm was used as coarse aggregate. The physical properties of the fine and coarse aggregates are summarized in Table 1.

**Table 1:** Physical properties of aggregates.

Aggregate	Dry-rodded density (kg/m^3^)	Water absorption (%)	Specific gravity	Maximum size (mm)
Fine aggregate	1600.0	0.14	2.57	4.75
Coarse aggregate	1660.50	0.75	2.88	19

**Fig. 1:**
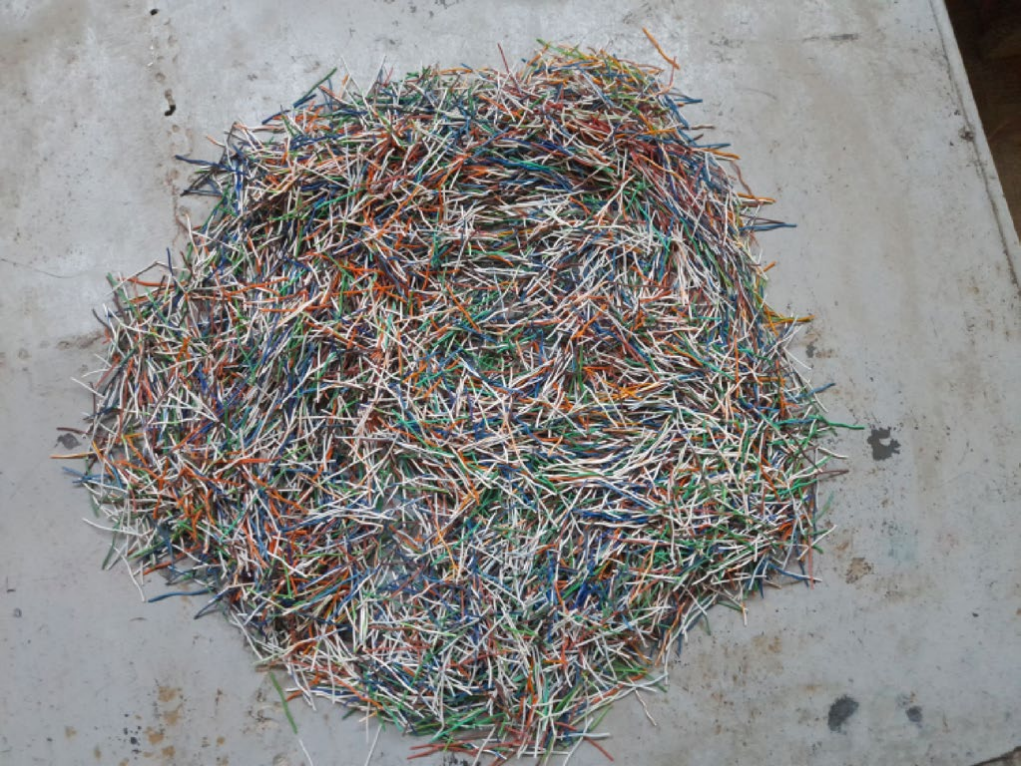
Electronic waste fibers.

To investigate the mechanical and dynamic performance of the composite, recycled PVC fibers were derived from discarded electrical cables. These cables consisted of an outer protective sheath and multiple internal copper conductors, each coated with PVC insulation. For this study, the outer sheath was removed, and the insulated conductors were utilized as the primary reinforcement. To maintain terminology consistent with the reinforcement’s interfacial behavior, the term “PVC fibers” is used herein for brevity and consistency to refer to these composite units, where the PVC insulation serves as the contact surface with the cementitious matrix while the copper core remains fully encapsulated. The fibers were manually sectioned into 30 mm and 50 mm lengths using high-precision cutters. The fibers’ nominal diameters were approximately 1 mm, verified using a digital caliper. To ensure experimental uniformity, any fibers exhibiting damaged insulation or irregular geometry were excluded. Prior to incorporation, the fibers were cleaned with a dry cloth to remove surface contaminants and stored under controlled laboratory conditions. The electronic waste fibers were incorporated as discrete fibers within the concrete matrix, as shown in Fig. 1.

### Mix design and specimen preparation

Concrete mix proportions were designed in accordance with ACI 211.1 guidelines to achieve a target compressive strength of 35 MPa. The optimized reference mix consisted of 450.24 kg/m³ of OPC, 764.47 kg/m³ of fine aggregate, 1022.2 kg/m³ of coarse aggregate, and 156.17 kg/m³ of water, corresponding to a water-to-cement ratio of approximately 0.35. This base mix design was consistently maintained across all specimens, as summarized in Table 2. Six fiber-reinforced concrete mixtures were prepared by incorporating recycled PVC fibers at 0.8%, 1.0%, and 1.2% by weight of cement, with fiber lengths of 30 mm and 50 mm. One additional mixture without fibers served as the control. The selected fiber contents were based on prior studies identifying optimal performance ranges for recycled plastic fiber-reinforced concrete.

**Table 2:** Mix the proportion of specimens.

Fine aggregate (kg/m^3^)	Coarse aggregate (kg/m^3^)	Cement (kg/m^3^)	Water (kg/m^3^)
764.47	1022.2	450.24	156.17

To ensure uniform fiber dispersion and minimize segregation, a manual dry mixing procedure was adopted. The recycled PVC fibers were first blended with the dry aggregates, followed by the gradual addition of cement and water. This sequential mixing process facilitated homogeneous fiber distribution throughout the concrete mix, contributing to consistent mechanical and dynamic performance across all mixtures.

**Fig. 2:**
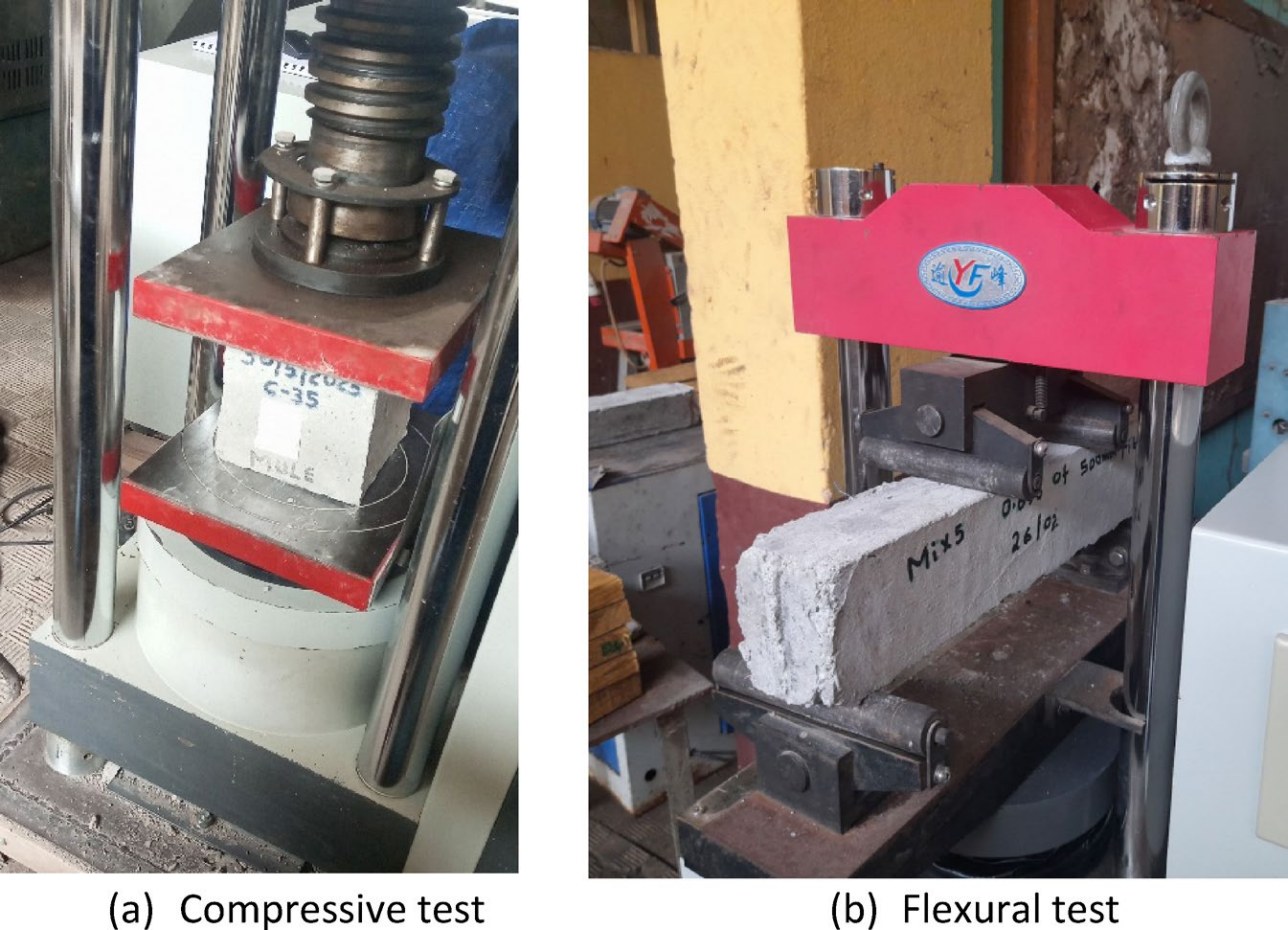
Mechanical behavior test setup.

Concrete specimens were cast following standard laboratory procedures. Cube specimens measuring 150 mm * 150 mm * 150 mm were prepared for compressive strength testing (Fig. 2a). Prismatic beams with dimensions of 100 mm * 100 mm * 500 mm were fabricated for four-point flexural testing, as shown in Fig. 2b. For damping testing, beam specimens with lengths of 500 mm, 700 mm, and 900 mm were produced, all with a uniform cross-section of 100 mm * 100 mm. The details of the specimen series are summarized in Table 3. For each mixture, three specimens were tested, and the reported strength values represent the average. The experimental variability was quantified using standard deviation (typically 0.43–0.6 MPa for compressive tests and 0.37–0.64 MPa for flexural tests), as illustrated by the error bars in the discussion figures. Specimens DB5 and DB6 were fabricated using the same concrete mix design as DB1; however, they were produced exclusively for free-vibration testing, and their compressive and flexural strengths were not experimentally measured.

Concrete was placed into molds and compacted using a vibrating table to ensure proper consolidation and minimize entrapped air. After demolding, all specimens were cured in a water tank for 28 days under controlled laboratory conditions at a temperature of (20 ± 6) ℃ and relative humidity of 96%.

**Table 3:** Test specimen series.

Specimen name	Beam length (mm)	Fiber content (%)	Fiber length (mm)	Compressive strength (MPa)	Flexural strength (MPa)
DB 0	500	–	–	35.57 ± 0.58	4.80 ± 0.37
DB 1	500	0.8	30	38.53 ± 0.55	4.90 ± 0.58
DB 2	500	0.8	50	36.13 ± 0.43	5.21 ± 0.54
DB 3	500	1.0	30	34.50 ± 0.50	4.95 ± 0.57
DB 4	500	1.2	30	33.93 ± 0.60	5.86 ± 0.64
DB 5	700	0.8	30	Not tested	Not tested
DB 6	900	0.8	30	Not tested	Not tested

### Damping test method

#### Free-vibration experimental setup

**Fig. 3:**
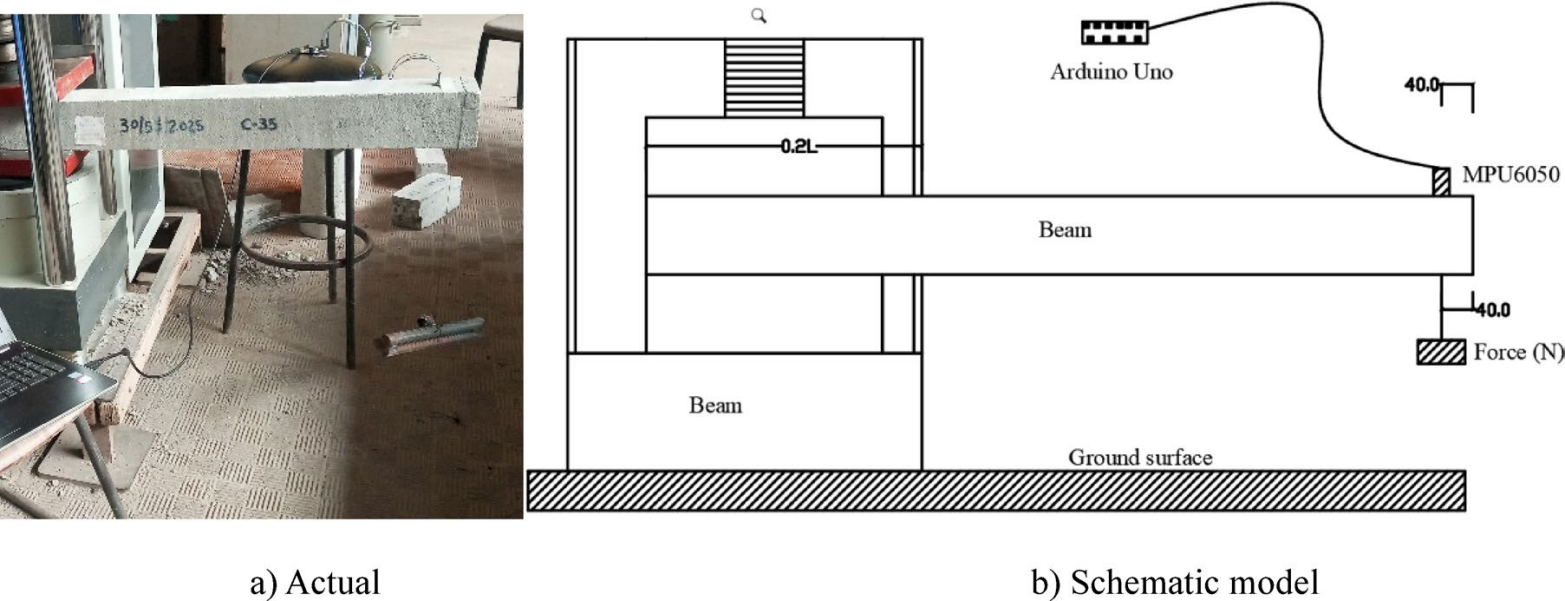
Damping test setup.

To evaluate the damping characteristics of concrete incorporating recycled PVC fibers, free-vibration attenuation tests were conducted using a cantilever beam configuration (Fig. 3a,b). One end of each beam specimen, corresponding to approximately 20% of the beam length, was rigidly clamped, while the opposite end remained free. This configuration follows established procedures commonly adopted in experimental studies on the dynamic response of concrete beams under free-vibration^7^^,32^.

A controlled excitation force was applied at the free end of the beam to generate an initial static deflection prior to release. Three excitation levels, corresponding to nominal forces of 20 N, 30 N, and 40 N, were employed to induce different initial vibration amplitudes. Immediately after release, the external excitation was removed, and the beam was allowed to vibrate freely under its own inertia and stiffness, as shown in Fig. 3. All tests were conducted under identical boundary conditions, instrumentation layout, and signal processing procedures to ensure consistency across specimens. The applied excitation force was varied to control the inertial vibration amplitude, thereby enabling the investigation of amplitude-dependent damping, a well-recognized characteristic of cementitious composites, rather than to characterize a force-dependent material property.

#### Instrumentation and data acquisition

The transient dynamic response of the beam was measured using an MPU6050 MEMS-based accelerometer mounted at the free end of the specimen to record vertical (Z-axis) acceleration. The sensor was interfaced with an Arduino Uno microcontroller, programmed to acquire acceleration data at 200 Hz over a 5-s recording window for each test. Before testing, the accelerometer was statically calibrated, and a gravitational offset of 9.82 m/s^2^ was applied to remove DC bias from the measured signal. Acceleration readings were converted to SI units using ±2 g sensitivity range (16384 LSB/g). The processed acceleration data were transmitted to a computer via serial communication for storage and subsequent analysis.

For each concrete mixture, two beam specimens were tested. To assess measurement repeatability and reduce random experimental variability, three independent free-vibration trials were conducted for each excitation level. The extracted damping ratios were averaged, and the corresponding standard deviations were reported to quantify experimental consistency.

#### Damping ratio extraction using a curve-fitting approach

The damping ratio of each beam was identified from the measured free-vibration acceleration response using a time-domain curve-fitting approach. The experimentally recorded signal was fitted to the analytical response of an underdamped single-degree-of-freedom (SDOF) oscillator, represented by a decaying sinusoid. Although a cantilever beam is inherently a multi-degree-of-freedom system, the free-vibration response within the selected analysis window was dominated by the first bending mode. Under this condition, the dynamic response can be reasonably approximated by an equivalent SDOF system to identify modal frequencies and damping. This assumption is commonly adopted in the experimental vibration studies of concrete and composite beams.

The governing equation of motion for an undriven damped SDOF system is expressed as:

1 $$m\ddot{x}+c\dot{x}+kx=0$$

Where *m*,* c*, and *k* represent mass, viscous damping coefficient, and stiffness of the system, respectively.

Dividing Eq. (1) by the mass *m* and introducing the undamped natural angular frequency ${\omega}_{n}$ and the damping ratio $\xi$, the equation can be written as:

2 $$\ddot{x}+2\xi{\omega}_{n}\dot{x}+{{\omega}_{n}}^{2}x=0$$

For an underdamped system $\left(0<\xi<1\right)$, the displacement response is expressed as:

3 $$x\left(t\right)=A{e}^{-\xi{\omega}_{n}t}\mathrm{cos}\left({\omega}_{d}t+\varphi\right)$$

Where $A$ is the initial amplitude, $\varphi$ is the phase angle, and *ω*_*d*_ is the damped natural angular frequency defined as:

4 $${\omega}_{d}={\omega}_{n}\sqrt{1-{\xi}^{2}}$$

Since the experimental measurements were recorded in terms of acceleration, the measured acceleration time history was fitted using an equivalent decaying sinusoidal representation of the form:

5 $$a\left(t\right)={a}_{max}{e}^{-\xi{\omega}_{n}(t-{t}_{o})}\mathrm{cos}\left({\omega}_{d}\left(t-{t}_{o}\right)+\varphi\right)$$

Where ${a}_{max}$ is the maximum acceleration amplitude, and *t*_*0*_ denotes the initial time of the free-vibration segment.

In the curve-fitting procedure, the parameters directly identified from the measured response were the acceleration amplitude ${a}_{max}$, damping ratio $\xi$, the damped natural angular frequency *ω*_*d*_, and phase angle *ϕ*. The corresponding undamped natural angular frequency *ω*_*n*_ can subsequently be obtained using Eq. (4).

For clarity and consistency, all frequencies reported in this study are expressed as the damped natural frequency in Hertz, defined as:

6 $${f}_{d}=\frac{{\omega}_{d}}{2\pi}$$

The frequency corresponds to the observable oscillation frequency of the decaying vibration response and is therefore used throughout the analysis and discussion of results.

#### Curve-fitting implementation and quality assessment

The curve-fitting procedure was implemented in Python using the scipy.optimize.curve_fit routine. The algorithm minimized the least-squares error between the measured acceleration time history and the analytical model given by Eq. (5). The fitted parameters included the maximum acceleration amplitude *a*_*max*_, the damping ratio *ξ*, the damped natural angular frequency *ω*_*d*_, and the phase angle *ϕ*. To ensure numerical stability and physically meaningful solutions, appropriate initial parameter estimates and bounds were imposed during the optimization process. The initial acceleration amplitude was estimated as the maximum absolute value of the processed acceleration signal, while an initial damping ratio of 0.02 was adopted based on typical empirical values reported for concrete beams.

To provide an objective justification for the SDOF approximation, a Fast Fourier Transform (FFT) was applied to each measured acceleration time history prior to curve fitting. The resulting spectra consistently exhibited a single dominant frequency peak, corresponding to the first bending mode of the cantilever beam, while higher-mode contributions were negligible within the selected free-vibration analysis window. The frequency associated with this dominant FFT peak was used solely to provide an initial estimate of the damped natural angular frequency *ω*_*d*_ for the optimization procedure. The FFT analysis, therefore, served both to verify the first-mode dominance and to provide a stable starting value for the curve-fitting algorithm.

Parameter bounds were applied to ensure physically meaningful solutions, with *a*_*max*_ ≥ 0, *ω*_*n*_ > 0, and 0 < *ξ* < 1, enforcing an underdamped response. The quality of each fit was assessed both visually and quantitatively using the coefficient of determination (*R*^*2*^). The consistently high *R*^*2*^ values obtained across all specimens indicate that the SDOF decaying sinusoidal model captures the dominant dynamic behavior of the tested beams. In addition, standard errors of the fitted parameters were obtained from the covariance matrix returned by the optimization routine, providing an estimate of uncertainty associated with the identified damping ratios. It should be noted that the identified damping ratios represent the global dynamic response of the beam-support-sensor system under the adopted experimental configuration, rather than purely intrinsic material damping properties.

## Results and discussion

### Concrete compressive strength

**Fig. 4:**
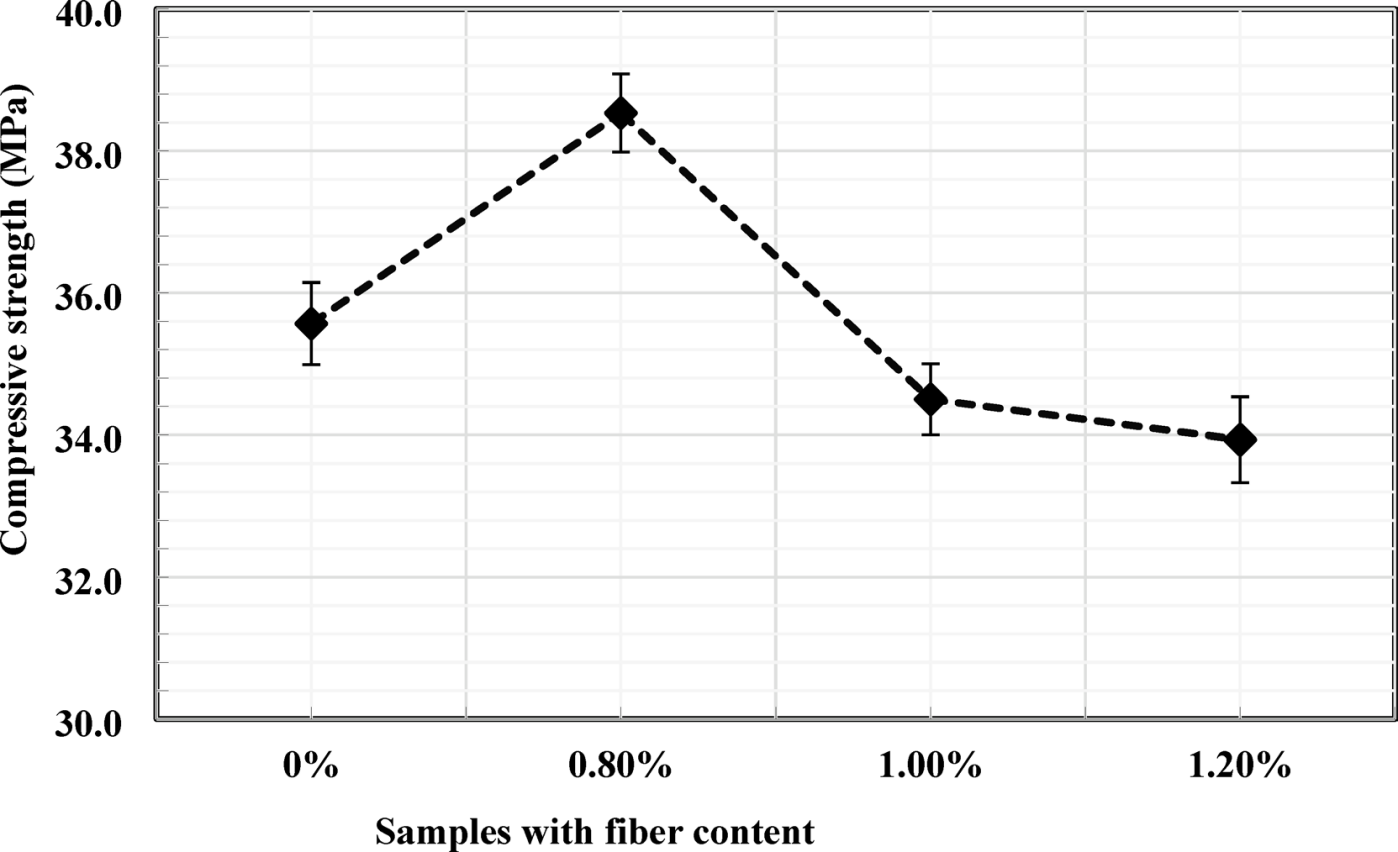
Effects of PVC fiber content on compressive strength.

The influence of PVC fiber content on the 28-day compressive strength of concrete is presented in Fig. 4. The control mix without fibers exhibited an average compressive strength of 35.57 MPa. Incorporation of 0.8% PVC fiber (by weight of cement) with a length of 30 mm increased the compressive strength to 38.53 MPa, representing an improvement of approximately 8.3% relative to the control mix. This enhancement may be attributed to the discrete fibers’ ability to bridge microcracks and delay crack initiation and propagation under compressive loading.

When the fiber content was increased to 1%, the compressive strength decreased to 34.50 MPa, corresponding to a reduction of approximately 3.10% relative to the control specimen. A further increase in fiber content to 1.2% resulted in compressive strength of 33.93 MPa, representing a 4.83% reduction compared to the control mix. The reduction in compressive strength at higher fiber dosages may be associated with reduced stress-transfer efficiency, resulting from localized fiber clustering and increased matrix heterogeneity. Similar reductions at elevated fiber dosages have been reported in previous studies, where non-uniform fiber dispersion and matrix discontinuities were identified as key contributing factors^33, 34, 35^. Previous investigations have also identified comparable optimal fiber ranges for PVC-reinforced concrete. For example, Gull and Balasubramanian^18^, reported a maximum compressive strength at a plastic fiber content of 1.0% with a fiber length of 30 mm, while Varghese and Boby^19^ identified an optimum dosage of 0.8% using 30 mm PVC fibers. Similarly, Islam et al.^36^, observed a peak compressive strength at approximately 1% PVC plastic fiber content with a fiber length of 20 mm. These results are broadly consistent with the present findings, which show that optimal compressive performance occurred at 0.8% fiber content with 30 mm fibers.

As illustrated by the narrow error bars in Fig. 4, the experimental scatter was limited, indicating good repeatability of the test results. The separation between the control and the 0.8% fiber dosage suggests that the observed increase in compressive strength is statistically significant.

**Fig. 5:**
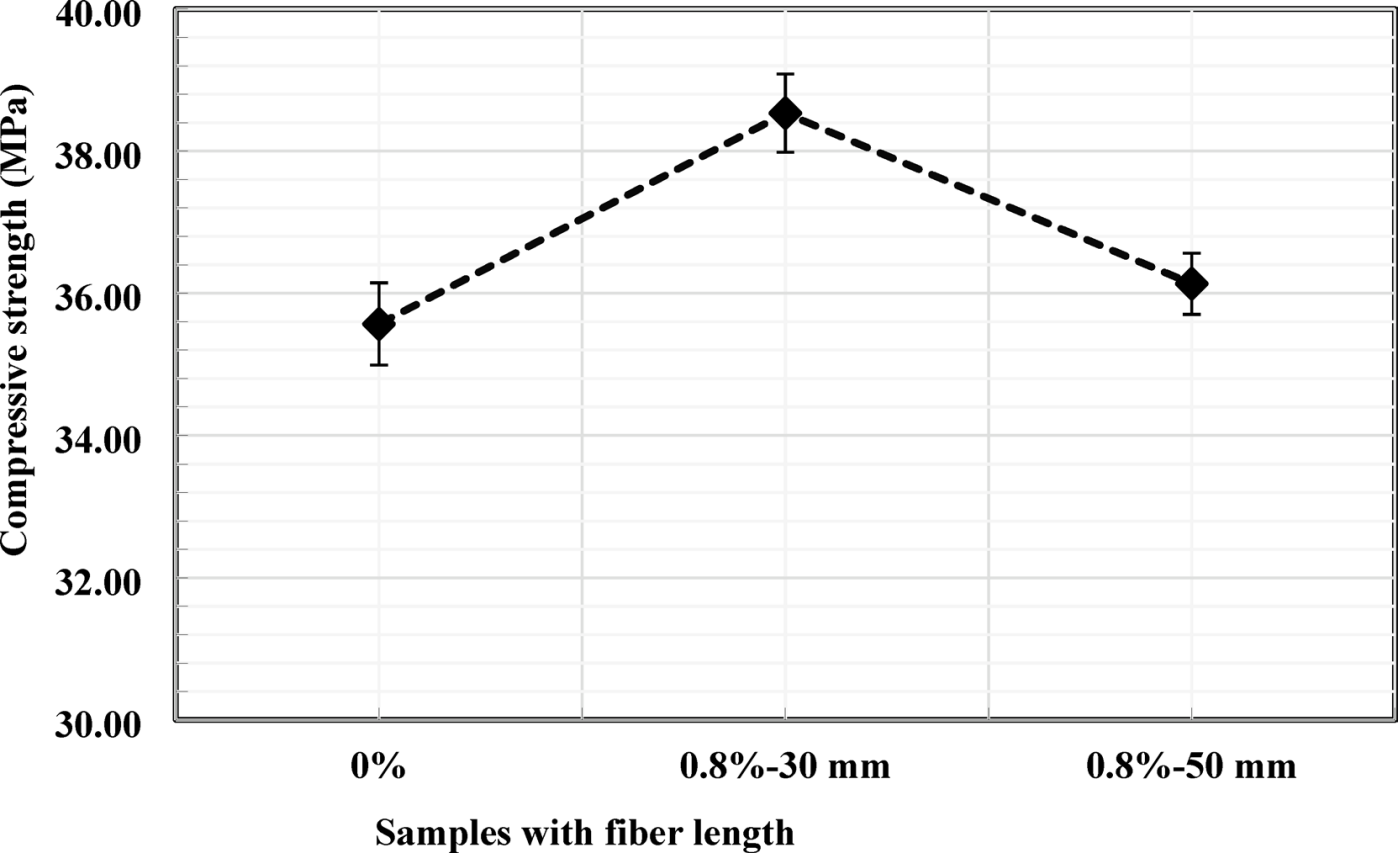
Effects of PVC fiber length on compressive strength.

The effect of PVC fiber length on compressive strength is illustrated in Fig. 5 for mixtures with a constant fiber content of 0.8%. The mixture incorporating 30 mm fibers achieved the highest compressive strength (38.53 MPa), whereas increasing the fiber length to 50 mm reduced the strength to 36.13 MPa, corresponding to a decrease of approximately 6.64%. This reduction may be related to differences in fiber distribution efficiency, as longer fibers can be more difficult to uniformly disperse within the matrix, potentially leading to localized stress concentrations and reduced load-transfer efficiency. In contrast, shorter fibers tend to distribute more uniformly, promoting a more homogeneous reinforcing network and improved compressive performance. The limited overlap of the error bars in Fig. 5 further supports the reliability of this trend.

### Flexural strength

**Fig. 6:**
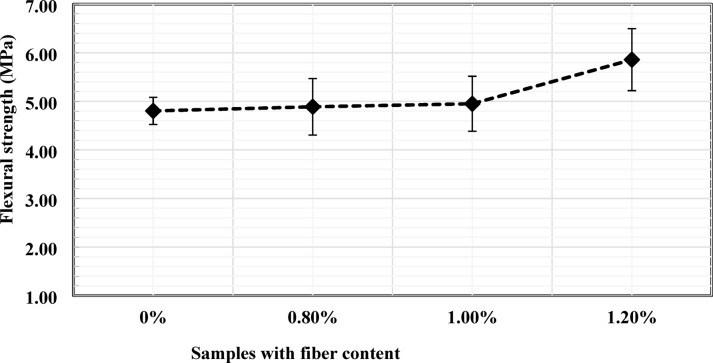
Effects of PVC fiber content on flexural strength.

The flexural performance of concrete mixtures incorporating PVC fibers was evaluated using four-point bending tests, as illustrated in Fig. 2. The influence of PVC fiber content on the 28-day flexural strength is summarized in Fig. 6. The control specimen without fibers exhibited an average flexural strength of 4.80 MPa. Incorporation of 0.8% PVC fibers with a length of 30 mm increased the flexural strength to 4.90 MPa, corresponding to an improvement of approximately 2.08% relative to the control. Increasing the fiber content to 1% further increased the flexural strength to 4.95 MPa, a 3.12% increase over the control.

A more pronounced improvement was observed when the fiber content was increased to 1.2%, with the flexural strength reaching 5.86 MPa, corresponding to an increase of approximately 22.1% compared to the control specimen. This progressive enhancement in flexural strength contrasts with the trend observed in compressive strength. The improvement can be attributed to the increased number of fibers bridging developing fiber cracks under bending, which enhances post-cracking load transfer and energy absorption. Similar improvements in flexural performance with increasing fiber dosage have been reported in previous studies. For example, Gull and Balasubramanian^18^ identified an optimal fiber content of approximately 1% and a fiber length of 30 mm to maximize flexural strength. In contrast, Islam et al.^36^, reported that although a fiber content of around 1% improved flexural performance, increasing fiber length beyond 20 mm did not provide additional benefits due to dispersion and workability limitations. Despite these variations, the present results are generally consistent with the literature, indicating that flexural strength is strongly influenced by fiber dosage through improved crack-resistance mechanisms.

Although the optimal fiber content in the present study is 1.2%, these results remain broadly consistent with previous findings, indicating that flexural performance generally improves with increased fiber dosage due to enhanced crack-bridging and energy-dissipation mechanisms.

The error bar distribution in Fig. 6 indicates that the improvements at 0.8% and 1% are relatively modest, whereas the increase at 1.2% represents a clearly distinguishable enhancement in flexural performance.

**Fig. 7:**
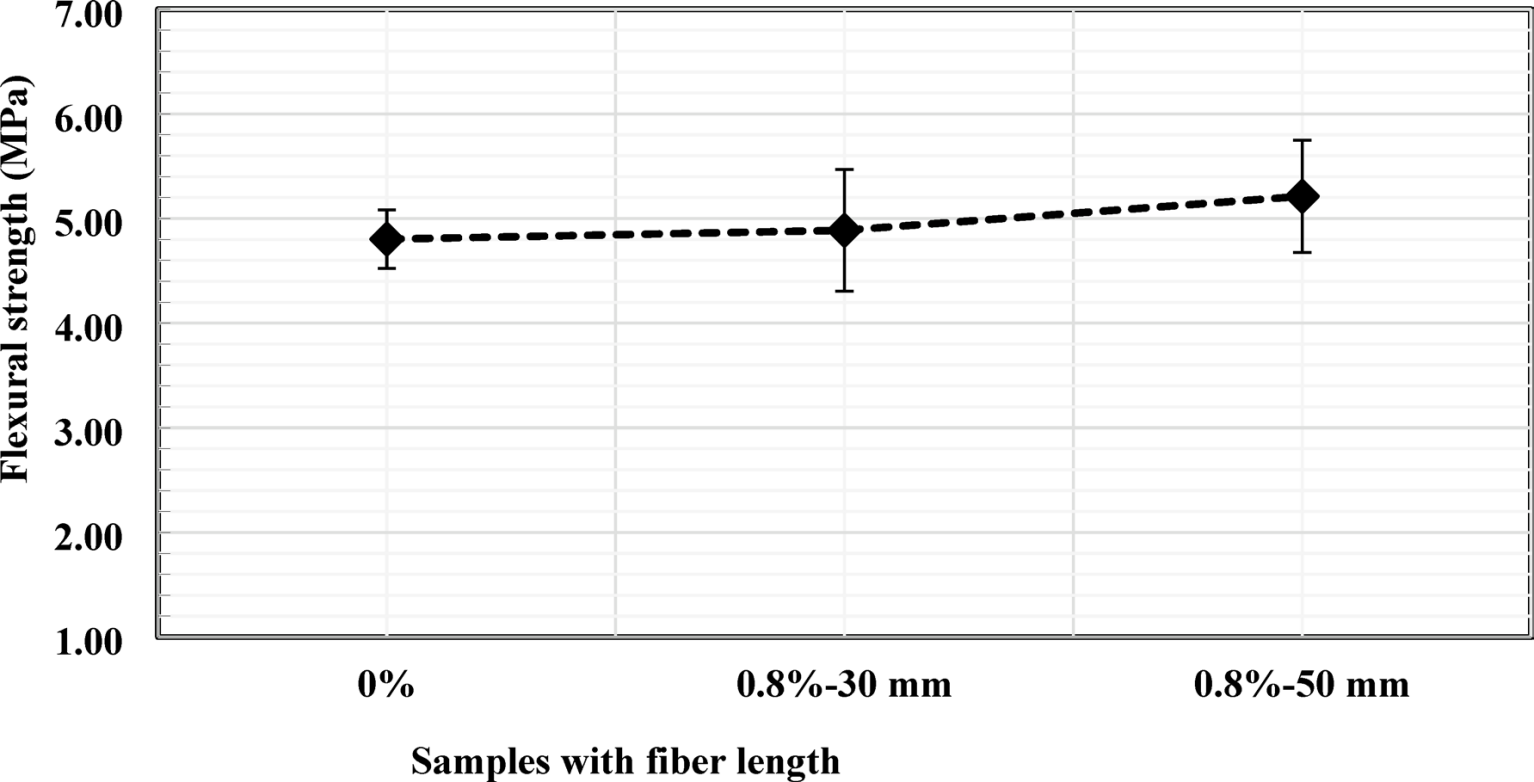
Effects of PVC fiber length on flexural strength.

The influence of fiber length on flexural performance is presented in Fig. 7 for mixtures with a constant fiber content of 0.8%. The specimen reinforced with 30 mm fibers achieved a flexural strength of 4.90 MPa, whereas increasing the fiber length to 50 mm resulted in a strength of 5.21 MPa, corresponding to an increase of approximately 8.54% relative to the control specimen and 6.32% relative to the 30 mm fiber mixture. In contrast to the compressive response, longer fibers appear more beneficial under flexural loading. The increased embedment length enhances fiber pull-out resistance, crack bridging capacity, and energy absorption during bending.

However, the improvement remains moderate, suggesting that fiber length alone is not the dominant parameter and that optimal flexural performance requires a balanced combination of fiber content, length, and workability. The partially overlapping error bars in Fig. 7 indicate that at a fiber dosage of 0.8%, the influence of fiber length is less pronounced than the effect of fiber content.

**Fig. 8:**
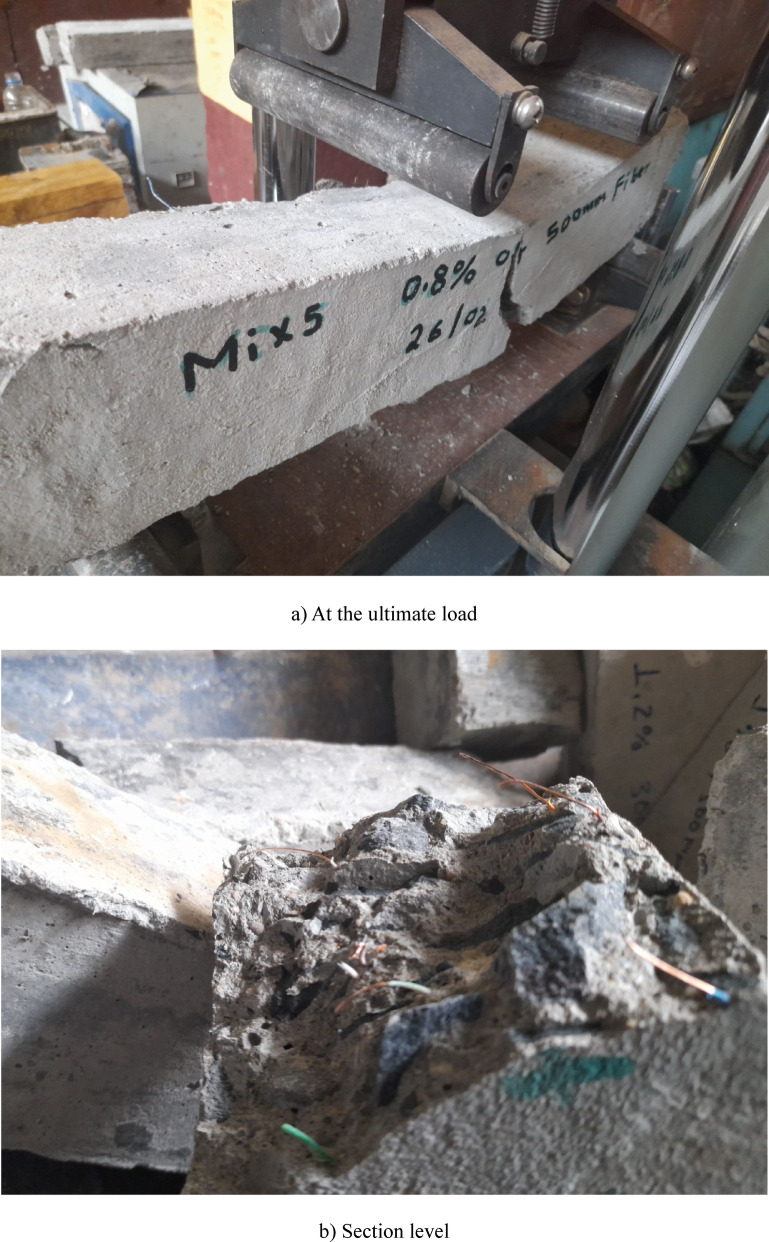
Flexural failure modes of PVC fiber-reinforced concrete beams.

The control specimen without fiber reinforcement exhibited a typical brittle failure under flexural loading, characterized by an abrupt crack formation and sudden separation into two distinct parts. In contrast, specimens incorporating PVC fibers demonstrated a more ductile failure behavior. As shown in Fig. 8a, crack initiation was delayed, and post-peak load degradation occurred gradually, indicating enhanced crack-bridging action and improved energy dissipation. Examination of the fractured section (Fig. 8b) revealed several fibers remaining partially embedded across the crack plane, with visible stripping of the PVC insulation from the copper core. This observation suggests significant interfacial friction and mechanical interlock between the fibers and the cementitious matrix, which contribute to resistance to crack propagation. The observed pull-out and debonding mechanisms confirm the effectiveness of recycled PVC fibers in enhancing post-cracking response and overall flexural toughness.

### Damping properties

#### Time-domain curve analysis

**Fig. 9:**
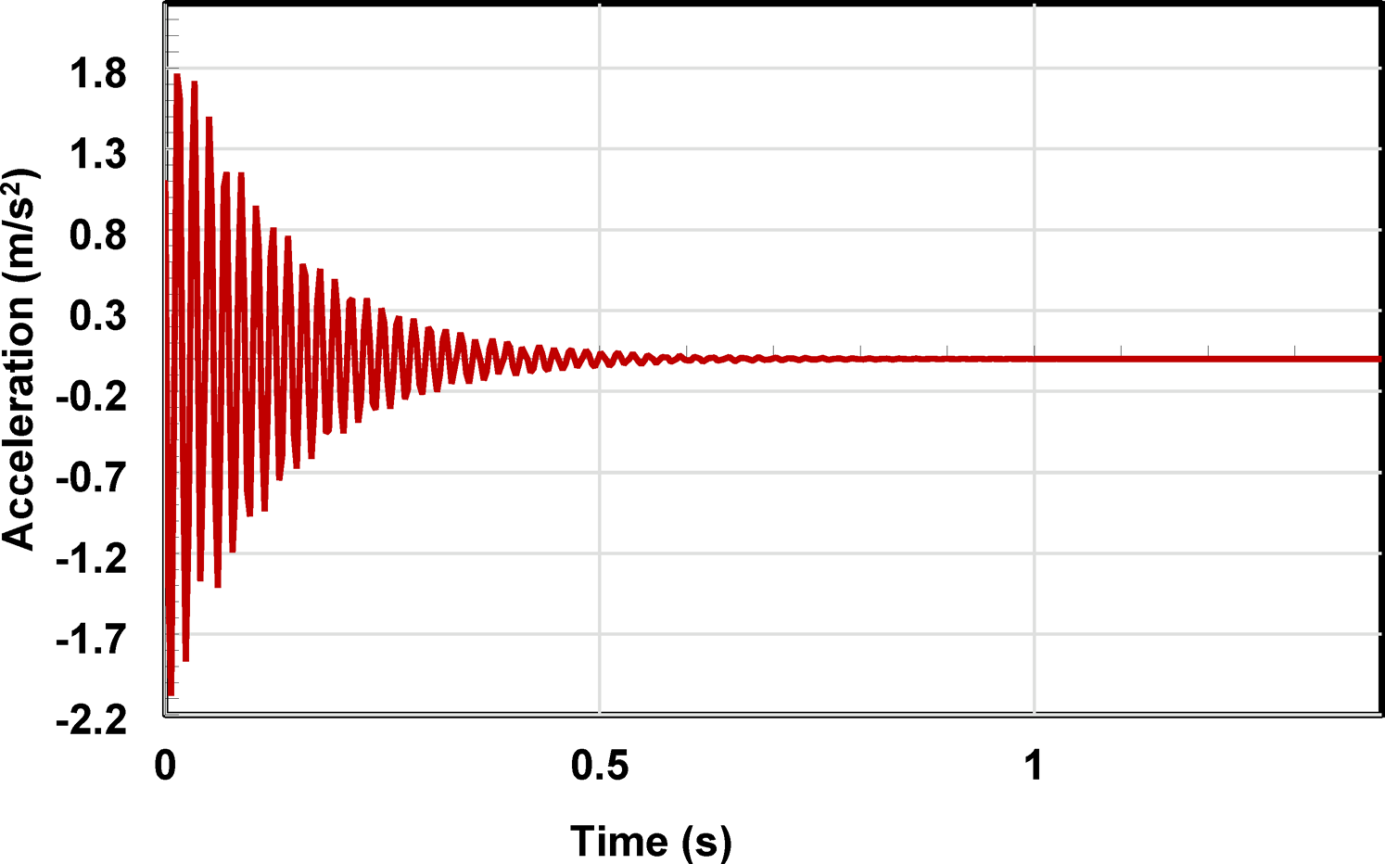
Acceleration vs. time plot using the curve-fitting method.

The experimentally identified dynamic parameters, including damping ratio, damped natural frequency, and goodness-of-fit metrics (*R*^*2*^), are summarized in Table 4. All values are reported as averages with corresponding standard deviations obtained from repeated free-vibration trials. The representative acceleration time history for specimen DB3 under a 20 N of excitation load is shown in Fig. 9. The recorded response exhibited a clear decaying oscillatory pattern, characteristic of an underdamped dynamic system dominated by the first bending mode.

The measured response was analyzed using the time-domain curve-fitting approach described in Sect. 2.3. A damped sinusoidal function was fitted to the acceleration signal, yielding an initial acceleration amplitude of 1.8 m/s^2^. The identified dynamic parameters for this case included a damping ratio of 2.33% and a damped natural frequency of 61.2 Hz. The coefficient of determination (*R*^*2*^) reached 91.12%, indicating good agreement between the analytical model and the experimental data. These results confirm the reliability of the adopted curve-fitting approach for characterizing the free-vibration response of the tested beams.

**Table 4:** Summary of the damping ratios and damped natural frequencies obtained from time-domain curve-fitting analysis.

Specimen name	Excitation load (*N*)	Damping ratio (%)	Damped natural frequency f_d_ (Hz)	*R* ^2^
DB 0	20	2.317 ± 0.051	63.197 ± 1.105	0.903 ± 0.023
	30	2.393 ± 0.045	61.627 ± 1.148	0.892 ± 0.035
	40	2.417 ± 0.070	59.917 ± 1.559	0.906 ± 0.036
DB 1	20	2.340 ± 0.100	62.533 ± 0.451	0.916 ± 0.028
	30	2.414 ± 0.112	61.267 ± 2.757	0.912 ± 0.040
	40	2.484 ± 0.207	59.803 ± 1.281	0.929 ± 0.032
DB 2	20	2.367 ± 0.131	61.967 ± 0.115	0.933 ± 0.023
	30	2.428 ± 0.139	60.767 ± 0.577	0.932 ± 0.015
	40	2.437 ± 0.110	59.767 ± 1.804	0.941 ± 0.015
DB 3	20	2.353 ± 0.107	62.413 ± 0.508	0.917 ± 0.016
	30	2.423 ± 0.121	61.433 ± 1.823	0.932 ± 0.009
	40	2.467 ± 0.125	58.867 ± 0.651	0.911 ± 0.044
DB 4	20	2.490 ± 0.118	62.241 ± 0.245	0.911 ± 0.052
	30	2.543 ± 0.122	60.367 ± 1.206	0.905 ± 0.031
	40	2.581 ± 0.129	59.167 ± 1.222	0.950 ± 0.027
DB 5	20	2.643 ± 0.246	54.567 ± 0.814	0.927 ± 0.035
	30	2.652 ± 0.271	52.453 ± 1.125	0.915 ± 0.023
	40	2.705 ± 0.113	50.217 ± 0.371	0.937 ± 0.025
DB 6	20	2.782 ± 0.053	42.967 ± 0.777	0.923 ± 0.042
	30	2.804 ± 0.118	39.867 ± 0.208	0.92 ± 0.04
	40	2.943 ± 0.232	35.867 ± 1.159	0.947 ± 0.015

#### The effects of fiber content and fiber length on measured damping ratio

**Fig. 10:**
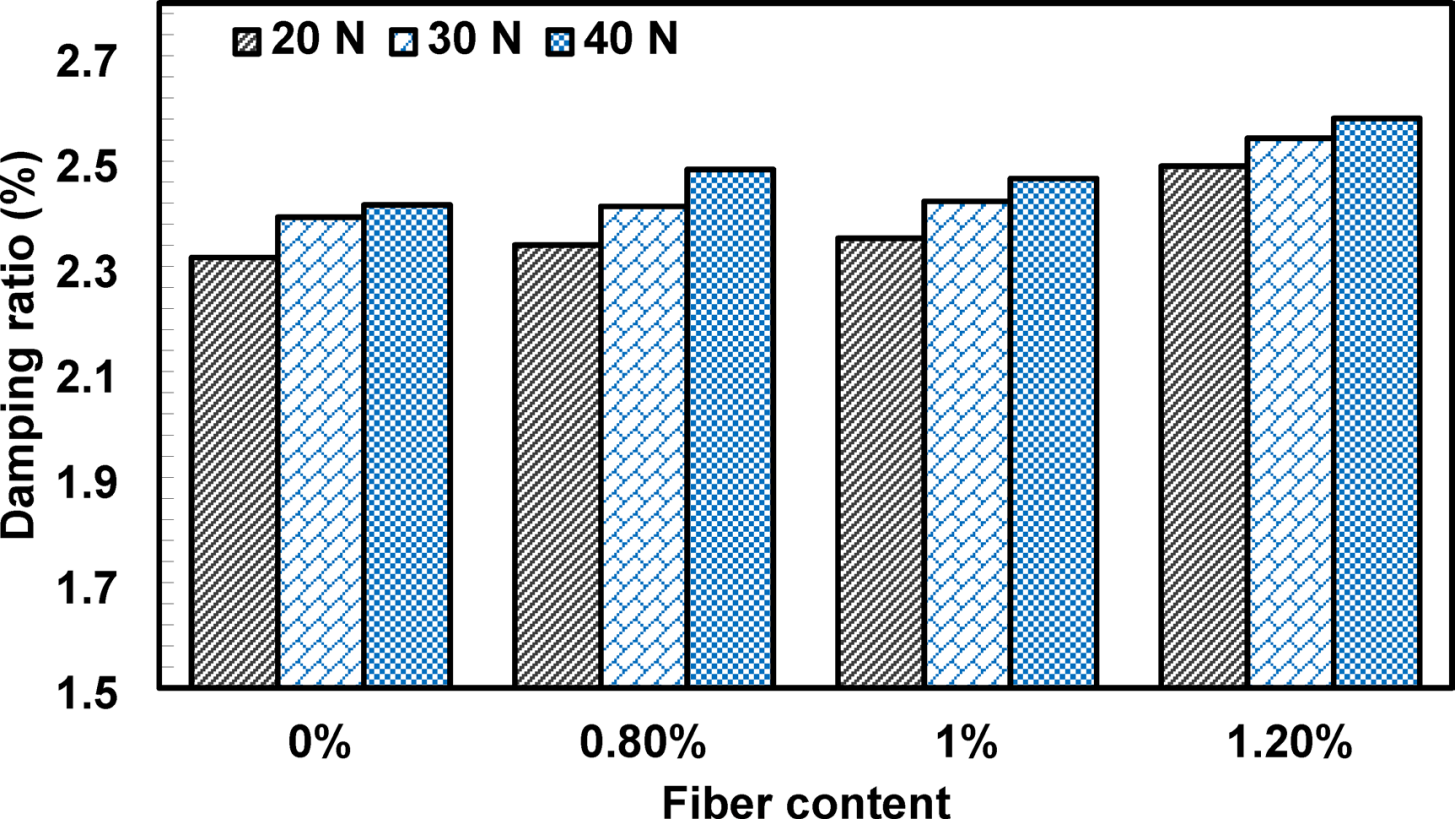
Effects of fiber content on the damping ratio of concrete.

The variation of the measured damping ratio with waste PVC fiber content is presented in Fig. 10. The results indicate that the measured damping ratio is influenced by both the applied excitation level, which governs the initial vibration amplitude, and the fiber content. For all mixtures, increasing the load from 20 N to 40 N resulted in a higher measured damping ratio, reflecting the well-established amplitude dependence of damping in concrete and cementitious composites. Similar trends have been reported by Liang et al.^37^, and Song et al.^38^, who observed increased damping ratios with increasing initial displacement levels. This amplitude-dependent behavior is generally attributed to enhanced internal friction and nonlinear energy dissipation mechanisms that become increasingly active at higher deformation amplitudes, including frictional sliding at microstructural interfaces and the initiation or propagation of microcracks, as discussed by Schaller and Fantozzi^39^ and Chung^40^. Concurrently, a modest decrease in the identified natural frequency was observed as the excitation level increased, which is consistent with the amplitude-dependent dynamic effects reported by Faizah and Aminullah^30^.

To clarify the influence of fiber content, the damping ratios are presented as relative changes from the control mixture, tested under identical excitation conditions. The incorporation of 0.8% PVC fiber led to only a marginal change in the measured damping ratio compared to the control. In contrast, increasing the fiber content to 1.0% resulted in a relative increase in damping of approximately 1.55%−2.2%, while a further increase to 1.2% fibers produced a more pronounced enhancement of about 6.27%−7.47% relative to the control mixture. These trends suggest that higher fiber content promotes greater energy dissipation during vibration. Similar improvements in damping capacity have been reported for other fiber-reinforced cementitious systems. For example, Zhou et al.^23^, observed that the combined use of an air-entrained agent and polypropylene fibers increased the damping ratio of concrete by approximately 5.26%. Likewise, Sofuoğlu et al.^21^, reported that the incorporation of recycled steel fibers into high-strength cementitious composites significantly enhanced damping performance, with increases of up to 200% relative to the reference mixture. In addition, Dehghanpour et al.^41^, demonstrated that hybrid fiber systems incorporating PVA, glass, and steel fibers can further improve the damping characteristics of ultra-high-performance concrete. Although the magnitude of improvement varies with fiber type, matrix composition, and test conditions, the approximately 7.47% increase observed in this study at a relatively low PVC fiber dosage indicates that recycled polymer fibers can provide meaningful energy dissipation while serving as a sustainable reinforcement.

**Fig. 11:**
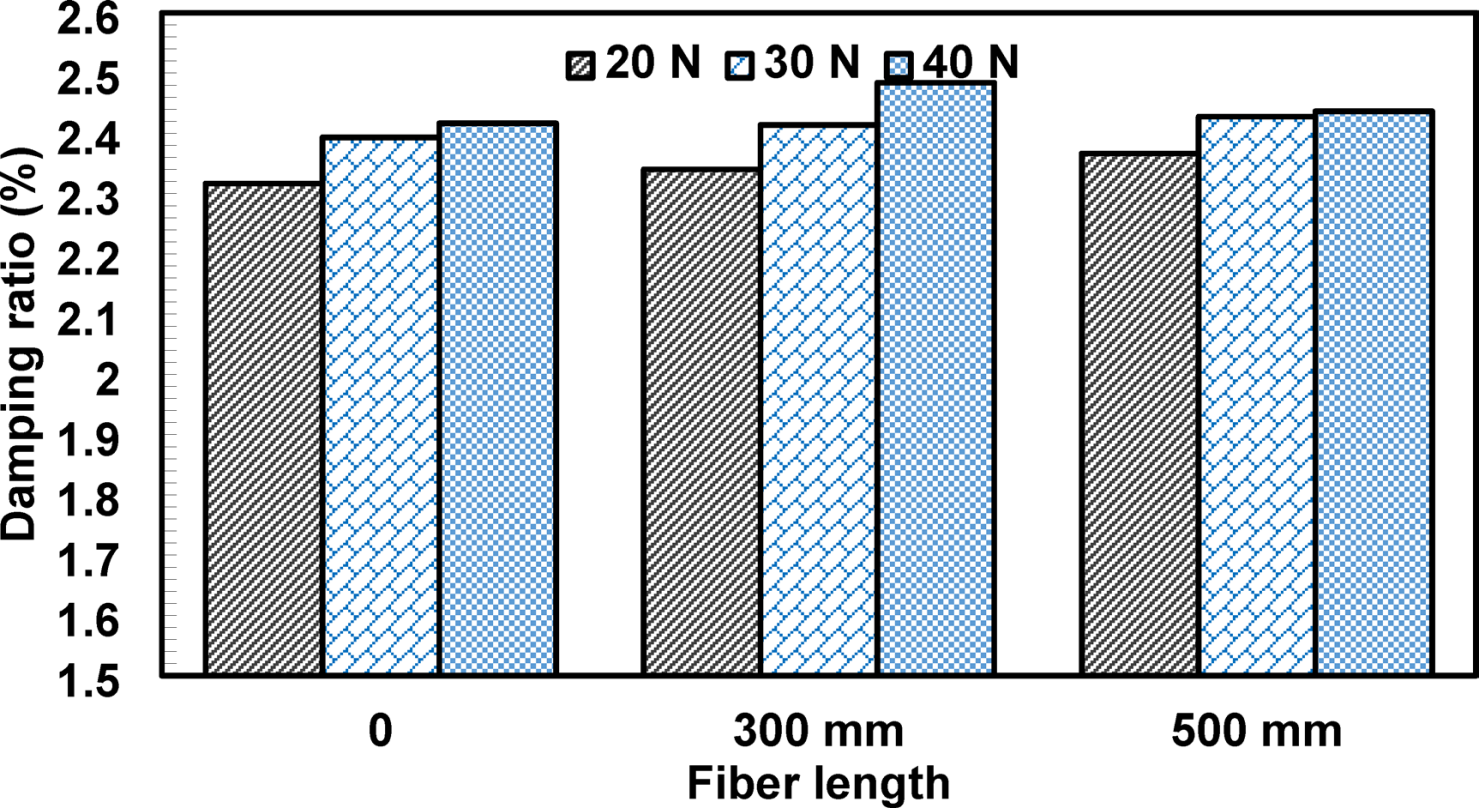
Effects of fiber length on the damping ratio of concrete.

The influence of fiber length on the measured damping ratio is illustrated in Fig. 11. Specimens incorporating longer fibers (50 mm) exhibited damping ratios approximately 0.83% to 2.16% higher than those with shorter fibers (30 mm). This slight increase may be associated with enhanced fiber-matrix interaction and increased interfacial contact area provided by longer fibers. Nonetheless, the effect of fiber length was considerably less pronounced than that of fiber content, indicating that fiber content plays a more dominant role in governing the damping response of the tested concrete beams.

#### Effects of beam length on measured damping ratio

**Fig. 12:**
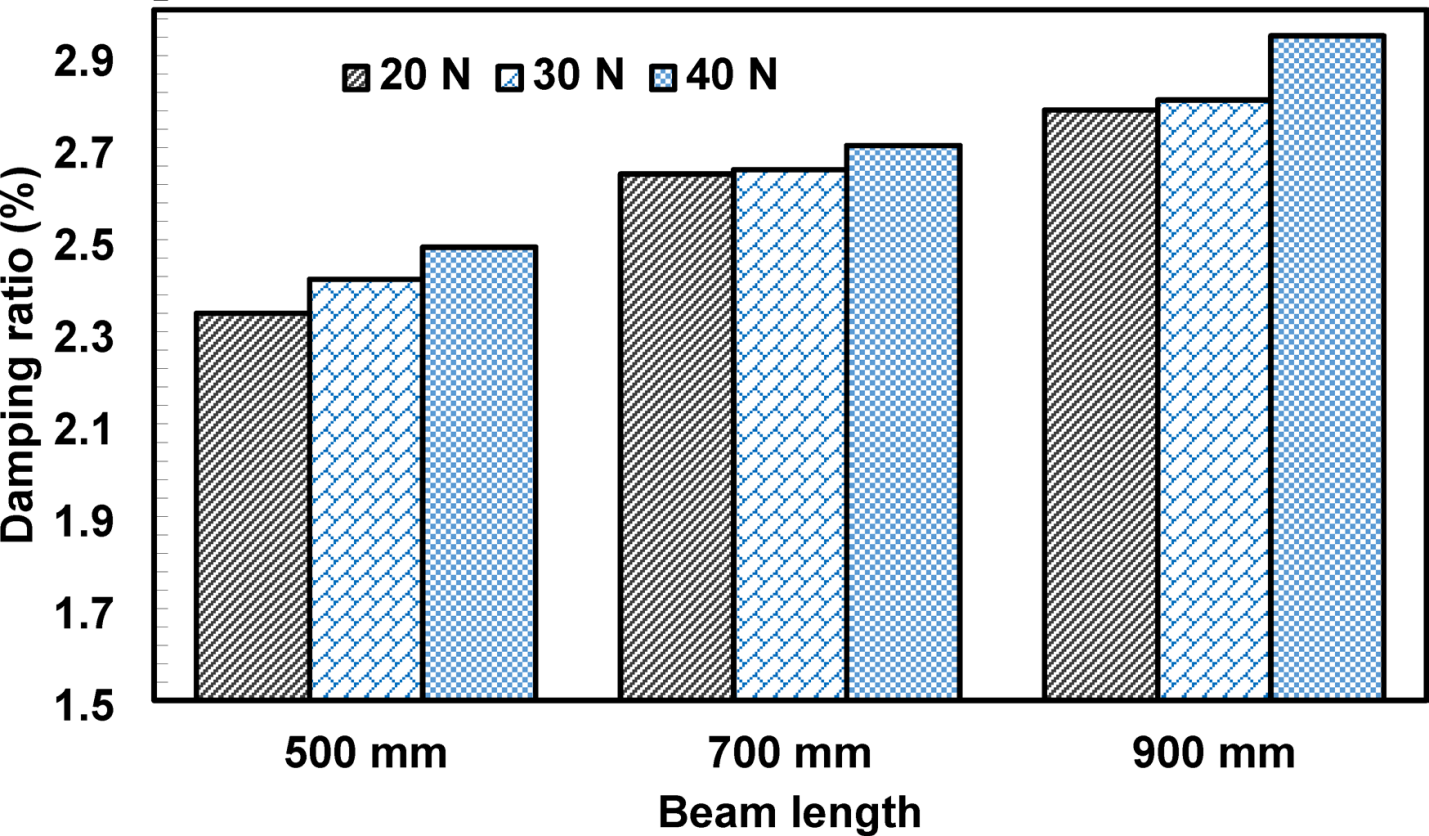
Effects of beam length on the damping ratio of concrete.

Figure 12 illustrates the variation of the measured damping ratio with beam length obtained from free-vibration tests. Beams with longer spans consistently exhibit higher damping ratios: compared with the 500 mm beams, the damping ratio increased by approximately 10.82–14.07% for the 700 mm beams and by 17.18–21.76% for the 900 mm beams. This length-dependent behavior reflects a system-level effect rather than a change in intrinsic material damping. While material damping arises from energy dissipation within the concrete matrix itself, primarily through microcrack friction and viscoelastic mechanisms, system-level damping encompasses additional contributions from the specimen’s geometry, boundary conditions, and global dynamic response. As previous studies have demonstrated, measured damping in free-vibration tests is sensitive to specimen geometry, boundary conditions, excitation method, and sensor location^30, 42, 48, 49^. Changes in beam length alter the specimen’s stiffness and modal characteristics, which in turn influence the contribution of distributed energy dissipation mechanisms, such as interfacial friction, micro-slip, and crack-related hysteresis, to the global dynamic response^43^. Accordingly, the observed dependence of the damping ratio on beam length should be interpreted cautiously within the limitations of the present test setup, recognizing that the reported values represent the response of the beam-support system rather than a purely intrinsic material property.

### Morphology

**Fig. 13:**
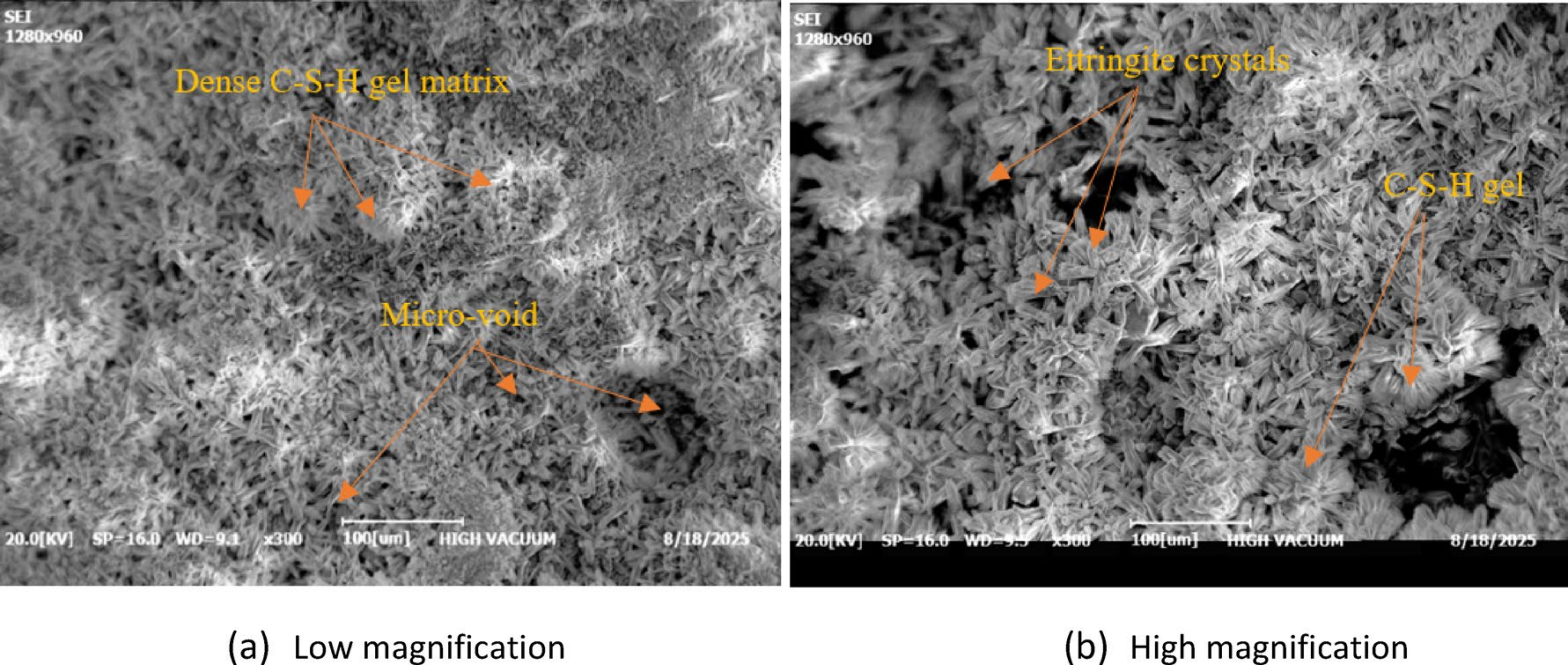
SEM image of cement matrix.

Scanning Electron Microscopy (SEM) was employed to examine the morphology of the cementitious matrix and the interfacial characteristics between recycled PVC fibers and the cement paste. SEM observations were conducted on fractured surfaces of cube compression specimens. Representative micrographs are shown in Fig. 13. The SEM images reveal a relatively dense and well-hydrated cement matrix, characterized by the presence of typical hydration products, including calcium silicate hydrate (C-S-H) gel and ettringite crystals (Fig. 13b). The observed microstructure indicates a well-developed hydration network. Isolated voids and micro-pores were also present (Fig. 13a), inherent features of cementitious composites that can influence their mechanical performance and dynamic response.

**Fig. 14:**
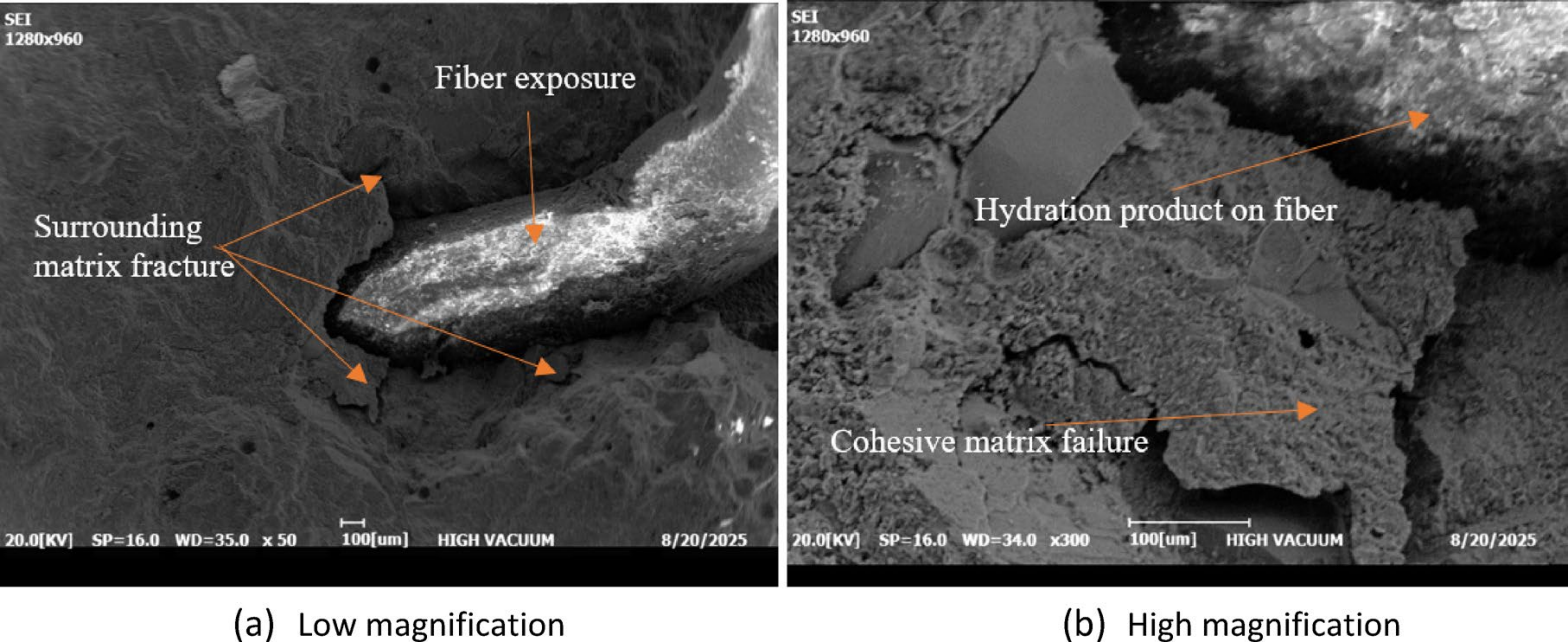
SEM images of the fiber-cement interface fractured surfaces.

Figure 14 illustrates the fiber-matrix interfacial region observed on fractured surfaces. At lower magnification (Fig. 14a), regions of fiber exposure and surrounding matrix fracture can be clearly identified, indicating that stress transfer between the cement paste and PVC fibers occurs primarily through interfacial bonding and mechanical interaction. This interfacial behavior contributes to improved post-cracking response, as reflected in the mechanical test results. At higher magnification (Fig. 14b), hydration products are observed adhering to the fiber surface, suggesting effective mechanical interlocking at the interface. The fracture morphology indicates cohesive failure of the surrounding paste in some regions, implying that the interfacial bond is locally comparable to the strength of the adjacent cementitious matrix.

**Fig. 15:**
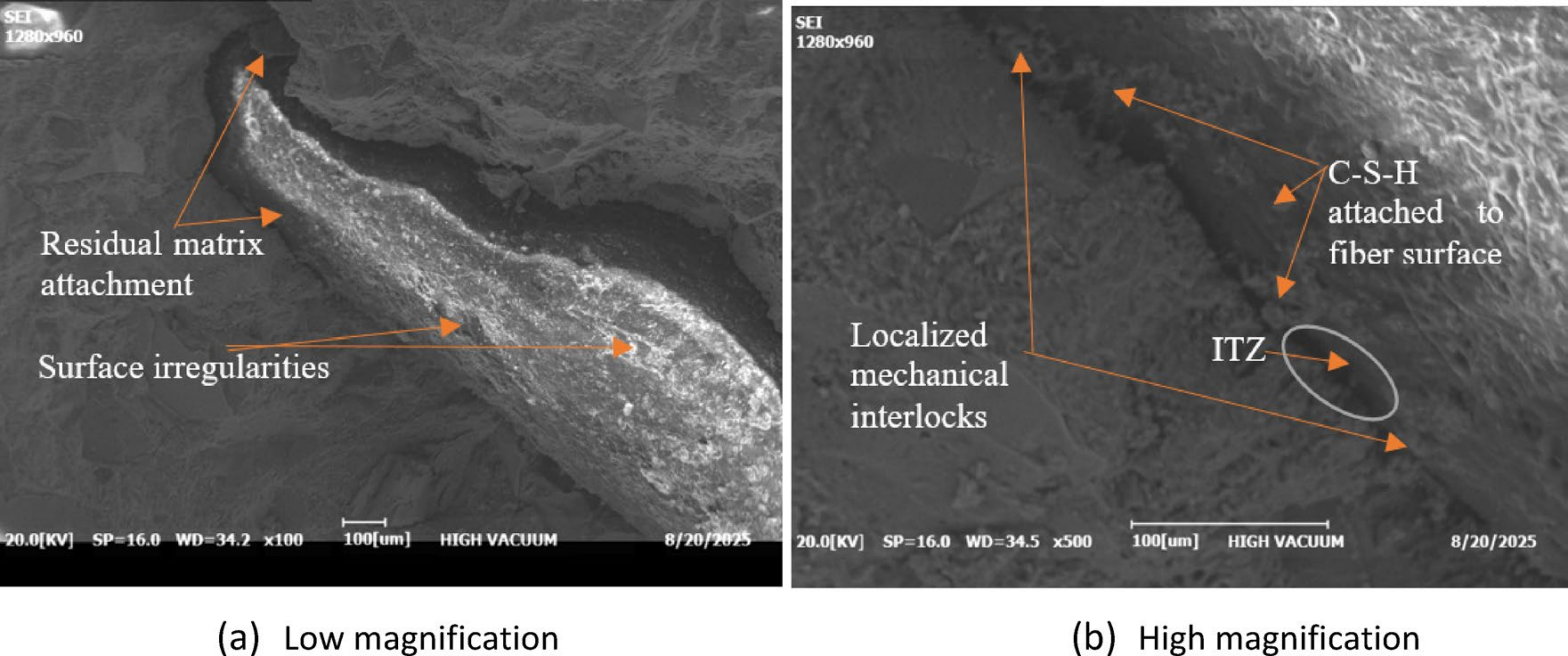
SEM images of the fiber-cement interface debonding and pull-out features.

Further insight into the fiber pull-out mechanism is provided in Fig. 15, which shows the interfacial transition zone (ITZ) at locations where partially extracted from the matrix. At lower magnification (Fig. 15a), the fiber surface exhibits noticeable surface irregularities and residual matrix attachment, indicating a combined failure mode involving interfacial debonding and partial fracture of the cement paste. At higher magnification (Fig. 15b), hydration products consistent with C-S-H morphology appear attached to the fiber surface, forming localized mechanical interlocks. These features enhance frictional resistance during fiber pull-out and improve the composite’s toughness and sustained load-carrying capacity.

The influence of fiber length on interfacial failure mechanisms can be inferred from the SEM observations. Longer PVC fibers provide a greater embedment length within the cementitious matrix, increasing the interfacial contact area and frictional resistance during pull-out^44^. Consequently, additional energy is required to overcome interfacial debonding and sliding during crack propagation, thereby enhancing energy dissipation under flexural loading through enhanced crack-bridging. Furthermore, the localized stripping of the PVC insulation from the copper core observed in Fig. 8b suggests a supplementary frictional mechanism that may further contribute to energy dissipation. In contrast, while shorter fibers (30 mm) promote a more uniform stress distribution within the matrix, their reduced embedment length limits the total pull-out energy that can be mobilized during crack opening.

Although SEM observations are inherently qualitative, the identified microstructural features, interfacial debonding, frictional sliding, and hydration product adhesion, are well-established contributors to energy dissipation. Prior research has demonstrated that these mechanics can be quantitatively characterized through single-fiber pull-out tests to determine bond strength and slip resistance, nanoindentation of the interfacial transition zone, and multiscale numerical frameworks linking interface properties to macroscopic damping behavior^45, 46^. Specifically, investigations using multiscale strategies have shown that the interaction between interfacial frictional resistance and matrix damage evolution governs the total energy loss during dynamic deformation^47^. Within this established theoretical framework, the observed PVC fiber-matrix interaction provides a plausible microstructural explanation for the enhanced damping characteristics. The identified features, interfacial debonding, frictional sliding, and hydration product adhesion, suggest that fiber engagement at the microscale contributes to the overall energy dissipation behavior of the composite.

### Limitations and future research directions

While this study provides insight into the mechanical performance and damping behavior of concrete incorporating recycled PVC fibers from electronic waste, several limitations should be acknowledged to contextualize the findings and guide future work. First, the reduction in compressive strength observed at higher fiber contents was attributed to possible localized fiber clustering and increased matrix heterogeneity. However, the SEM observations presented offer only qualitative, localized insight into fiber-matrix interactions and do not provide a statistically representative assessment of global fiber dispersion. Future studies should therefore employ quantitative dispersion characterization techniques, such as image-based dispersion indices, three-dimensional X-ray computed tomography, or automated image analysis methods, to establish a more rigorous microstructure-property correlation.

Second, the damping properties in this study were evaluated solely through a free-vibration test. While free-vibration decay provides a fundamental measure of system-level energy dissipation and is relevant to vibration amplitude control under service-level dynamic actions such as traffic or wind, it does not capture frequency-dependent or load-specific damping mechanisms. Moreover, the measured damping reflects combined material and structural contributions associated with specimen geometry and boundary conditions. Future investigations should adopt experimental strategies capable of isolating intrinsic material damping, such as forced-vibration or harmonic excitation tests over a range of frequencies, specimen testing with varying geometries but identical material composition, and inverse analysis or finite-element model updating.

Despite these limitations, the results establish a clear baseline for assessing the mechanical enhancement and vibration-damping potential of recycled PVC fiber-reinforced concrete. The outlined research directions provide a pathway to improve damping characterization and strengthen the engineering applicability of electronic-waste-derived fiber systems in sustainable construction materials.

## Conclusion

This study investigates the influence of recycled PVC fiber content and length on the mechanical and dynamic behavior of concrete through compressive and flexural strength tests and free-vibration experiments, supported by SEM microstructural observations. Based on the experimental results, the following conclusions can be drawn:

- An optimal PVC fiber content of 0.8% produced the highest compressive strength. Increasing the fiber content up to 1.2% resulted in a slight reduction in compressive capacity, indicating that excessive fiber incorporation may introduce matrix heterogeneity that affects compressive performance.
- Flexural performance improved progressively with increasing fiber content, with a maximum enhancement of 22.1% observed at 1.2% fiber content compared with plain concrete. Longer fibers (50 mm) provided an additional 6.33% increase in flexural strength relative to the 30 mm fibers, attributed to more effective crack bridging across wider flexural cracks.
- Fiber-vibration testing indicated that incorporating 1.2% PVC fiber produced the highest relative increase in the damping ratio (up to 7.47%) compared to the control mix. Fiber length had a measurable but secondary influence on damping, with longer fibers consistently producing slightly higher damping ratios than shorter fibers, although the magnitude of this effect was smaller than that associated with fiber content.
- Variations in beam length affected the measured damping response, reflecting system-level dynamic behavior associated with specimen geometry and boundary conditions rather than intrinsic material damping alone.
- SEM observations revealed effective interaction between PVC fibers and the cementitious matrix, including hydration products adhering to fiber surfaces and evidence of interfacial debonding and controlled pull-out. These microstructural features support the enhanced crack bridging, energy dissipation, and improved post-cracking behavior observed in the mechanical and dynamic tests.

Overall, the results demonstrate the potential of recycled PVC fibers derived from electronic waste as a sustainable reinforcement for improving the mechanical performance and free-vibration response of concrete. From a practical perspective, incorporating recycled PVC fibers offers a promising approach to enhancing concrete performance while promoting the reuse of electrical cable waste. The experimental results indicate that at moderate dosages (up to approximately 0.8%), these fibers can enhance flexural strength and vibration damping without causing significant reductions in compressive strength. Such characteristics may be particularly beneficial for applications where crack control, impact resistance, and vibration mitigation are important, including industrial floor slabs, machine foundations, pavements, and other elements subjected to dynamic loading.

## Data Availability

The datasets used and/or analysed during the current study available from the corresponding author on reasonable request.
